# Business and Human Rights Regulation After the UN Guiding Principles: Accountability, Governance, Effectiveness

**DOI:** 10.1007/s12142-022-00656-2

**Published:** 2022-03-14

**Authors:** René Wolfsteller, Yingru Li

**Affiliations:** 1 Department of Political Science, Martin Luther University Halle-Wittenberg, Halle (Saale), Germany; 2 Adam Smith Business School, University of Glasgow, Glasgow, UK

**Keywords:** UN Guiding Principles on Business and Human Rights, Business and human rights regulation, Corporate accountability, Polycentric governance, Due diligence, Norm diffusion

## Abstract

Since the UN Guiding Principles on Business and Human Rights (UNGPs) were adopted by the UN Human Rights Council in 2011, they have diffused into policy frameworks, laws, and regulations across the globe. This special issue seeks to advance the interdisciplinary field of human rights research by examining key elements of the emerging transnational regime for the regulation of business and human rights. In seven original contributions, scholars from political science, law, accounting, and philosophy critically reflect on the theoretical foundations of the UNGPs, they analyze the effectiveness of implementation mechanisms and current regulatory practice, and they advance proposals for the future development of the business and human rights regime. In this introduction, we prepare the ground for these analyses, proceeding in three steps. Firstly, we argue that the adoption of the UNGPs has triggered a norm cascade which requires a distinctive, empirically oriented research agenda focusing on the scope, governance, and effectiveness of corporate human rights accountability norms and instruments. Secondly, we explain how the articles in this special issue contribute to that research agenda by addressing these themes. Thirdly, we provide an overview of the individual contributions and point out avenues for future research.

## Introduction

Following the endorsement of the Guiding Principles on Business and Human Rights (UNGPs) by the UN Human Rights Council (UNHRC) in 2011, they have become widely regarded as “the most authoritative statement of the human rights duties or responsibilities of states and corporations adopted at the UN level” (De Schutter 2013: xvii).^1^ Developed in a worldwide consultation process by the Special Representative of the UN Secretary-General (SRSG), John Ruggie, the UNGPs were aimed at tackling and preventing business-related human rights abuses by closing governance gaps in the largely unrestrained global market economy (Ruggie 2013: xxiii).

Although many corporations contribute positively to realizing human rights, e.g., through job creation, the production and distribution of goods, services, and infrastructure, business activities also impact negatively on human rights. Some of the most severe and widespread forms of corporate human rights abuse include the dislocation of indigenous communities without compensation or consultation, the impairment of people’s health and safety due to unfit working conditions and destruction of the environment, the leaking of individuals’ data to government agents, the denial of freedom of expression and of association, discrimination and sexual harassment at the workplace, and sweatshops, bonded labor, and child labor in the transnational supply chains of global brands (see Bernaz 2017: 1–2; Ruggie 2013: xv-xvi; ILO 2017). However, it is rather difficult to hold business enterprises to account for human rights abuses, for various reasons.

Firstly, despite noticeable shifts in the attitudes of corporate leaders and policymakers over the last 30 years, a broad responsibility for the protection and realization of human rights within the corporation’s sphere of influence is still far from being universally accepted, and there is a persistent unwillingness among governments to introduce more stringent regulations for the corporate sector (Ruggie 2013: xxv; see also Nowrot 2018: 23–34).

Secondly, voluntary initiatives for corporate self-regulation, such as the UN Global Compact and other Corporate Social Responsibility (CSR) schemes, are unable to fill the accountability gap as they do not generate transparent, legally binding obligations, nor do they guarantee access to effective remedies for victims of corporate human rights abuse. CSR research suggests that companies voluntarily committing themselves to respect human rights tend to be highly selective in their engagement with rights norms and that substantial behavioral change is rare (LeBaron et al. 2021; Favotto and Kollman 2022).^2^

Thirdly, the international human rights regime is primarily designed to protect human rights from interference by state actors. The UN treaty regime imposes direct legal obligations only on states and applies to private businesses rather indirectly and in a very limited way.^3^ Moreover, not all states have ratified the key UN treaties or are willing to implement them (including the USA and China).^4^ A UN initiative to establish a mandatory code of conduct with direct human rights obligations for transnational enterprises under international law (the “UN Norms”)^5^ failed to secure the support of western governments, the business community, and the UN Commission on Human Rights in 2004 (see Hamm 2022; Ruggie 2020: 71).

Fourthly, in the absence of a centralized and effective international regulatory body or system, corporate human rights accountability is regulated by a patchwork of national and international rules, laws, and standards with differing degrees of bindingness and enforceability, often dealing only with specific rights norms and/or types of companies within a particular state or region.^6^

Fifthly, although transnational corporations (TNCs) account for half of global exports, one-third of the global GDP, and approximately one-quarter of global employment (OECD 2018), their liability is limited under public (national and international) law because parent companies and their subsidiaries and suppliers operating in different jurisdictions are treated as separate legal entities. Due to this “separation principle,” it is extremely difficult, often impossible, for victims to hold the parent company to account for human rights abuses committed by subsidiaries or suppliers and to seek legal redress (Krajewski 2018; Schilling-Vacaflor 2021).

When John Ruggie was appointed as Special Representative on human rights and transnational corporations and other business enterprises in 2005 by then UN Secretary-General Kofi Annan, his mission was to find a way out of this impasse by exploring the potential for a new regulatory framework. Ruggie and his team identified three corner stones (or “pillars”) of such a framework: (1) the state duty to protect against human rights abuse, including by third parties such as business corporations; (2) the corporate responsibility to respect human rights; and (3) the provision of access to effective remedies for victims of business-related human rights abuse.^7^ After intense consultations with stakeholder groups from all over the world, Ruggie specified the “Protect, Respect and Remedy” framework through a set of 31 Guiding Principles on Business and Human Rights^8^ which was unanimously endorsed by the UN Human Rights Council on 16 June 2011.^9^

Despite persistent critique of the form and substance of the UNGPs by scholars and civil society actors (e.g., Rodríguez-Garavito 2017; Mares 2012; Deva and Bilchitz 2013), the UNHRC’s endorsement of the UNGPs and their support by key actors has triggered a remarkable process of the diffusion of corporate human rights accountability norms across public, civil, and corporate governance systems. Over the past 10 years, the UNGPs have been integrated into the policy frameworks and guidelines of organizations such as the European Union^10^ and the OECD,^11^ and leading TNCs.^12^ They have inspired investment regulations for different sectors of the globalized market economy,^13^ and they have encouraged the development of national systems for the regulation of corporate human rights conduct through instruments such as National Action Plans on business and human rights,^14^ National Contact Points,^15^ and due diligence laws.^16^ Although the latest draft of a future international binding treaty on business and human rights––which was initiated by the UNGPs’ critics and has been in the making under the auspices of a UN working group since 2015^17^––departs from the approach of the UNGPs in important respects, it, too, builds on their concepts and standards, such as due diligence.^18^

While a fast-growing body of literature seeks to keep pace with that “norm cascade” (Finnemore and Sikkink 1998: 902) and the regulatory innovations in the field, business and human rights scholarship has been dominated so far by a legal perspective, focusing the debate on issues of legitimacy and on the advantages and drawbacks of soft law and hard law instruments (Ratner 2020; Krajewski 2018; Bernaz 2017; Nolan 2013). Important as they are, these debates do not fully comprehend the empirical complexity of the business and human rights regime, nor the dynamics of the norm diffusion processes underlying its manifestation.

We still know relatively little about how far and how deep corporate human rights accountability norms have spread across different governance systems. It remains an open question whether the responsibilities to respect and protect human rights can (and should) be translated into business principles so as to find broader acceptance, and whether the aim of realizing human rights can, in fact, be integrated into business practice in ways that are compatible with corporate profit-seeking in global market capitalism at all.^19^ In addition, which structural and cultural conditions hinder or facilitate the effective implementation of tools and strategies for advancing human rights due diligence and access to remedies for victims? Most strikingly, we still know little about the extent to which business practices have changed in response to different regulatory initiatives and increasing stakeholder expectations towards corporate human rights accountability.^20^ Various scholars have advised caution as to the unintended consequence of the diffusion of corporate human rights accountability norms having transformed business actors into norm entrepreneurs who may try to co-opt the human rights regime and discourse (e.g., Deva 2020; Scheper 2019).

Addressing these questions requires a distinctive, empirically oriented research agenda to critically examine the scope and construction, the governance, and the effectiveness of corporate human rights accountability norms across different governance systems. In the following section, we argue that the concepts and insights of norm diffusion scholarship are particularly useful for the interdisciplinary analysis of the business and human rights regime, and we explain how this special issue contributes to that research agenda. We then outline the core arguments of the individual articles before we conclude by pointing out avenues for future research.

## Accountability, Governance, Effectiveness: Exploring the Diffusion of Human Rights Norms

Drawing inspiration from the rich body of norm scholarship which has emerged since the 1990s in the field of International Relations and beyond (e.g., Finnemore and Sikkink 1998; Risse et al. 1999; Acharya 2004; Wiener 2004; Sandholtz 2008; Zimmermann 2016), we propose broadening the focus of business and human rights research from legal technicalities to the full spectrum of what scholars have called the norm “life cycle” (Finnemore and Sikkink 1998: 892). Based on an understanding of norms as intersubjective “standard[s] of appropriate behavior for actors with a given identity” (Finnemore and Sikkink 1998; 891; Stimmer 2019: 271), we define corporate human rights accountability norms as collective expectations of a corporation’s adequate answerability for its human rights conduct to the stakeholders affected by its operation. Focusing on the norm life cycle means examining the processes of their strategic social construction and diffusion by norm entrepreneurs, their acceptance and internalization by key actors and organizations, and their transformation and contestation (Finnemore and Sikkink 1998; Risse et al. 1999; Stimmer 2019).

In contrast to the rather mechanistic assumptions about the efficacy of the law in large parts of legal scholarship, empirical research on transnational processes of norm diffusion has produced a critical awareness among norm scholars that the establishment and diffusion of a norm may not automatically lead to its effective implementation or to the adaptation of the behavior of relevant actors as intended by the norm constructers. Norm scholars differentiate between “alternate endings of the norm life cycle” (Stimmer 2019: 270), pointing to different degrees of normative agreement and norm stability. In so doing, they usefully distinguish between the acceptance or contestation of norm frames (discursive justifications), on the one hand, and of norm claims (the substance of a norm requiring specific actions), on the other hand (Stimmer 2019: 270). In addition, the norm frame and/or claim may be transformed and significantly altered through the appropriation and translation of norms into the local context in strategic processes of their social reconstruction (see Wiener 2004; Zimmermann 2016; Wolfsteller 2020).

Adopting the lens of norm diffusion for the analysis of the transnational business and human rights regime offers the analytical advantage of furthering our empirical understanding of (1) the scope and construction of corporate human rights accountability norms, (2) the modes of their governance, and (3) the conditions for their effectiveness.

### The Scope and Construction of Corporate Human Rights Accountability

Foregrounding the *construction, dissemination and transformation* of norms, norm scholarship can inspire business and human rights research to examine the scope and meaning of corporate human rights accountability; to analyze which human rights accountability norms have emerged and diffused (and why); which alternative understandings of human rights accountability have been superseded in these processes; and how the constructed norms were transformed in their diffusion and implementation. The contributions in this special issue address these questions by analyzing the creation and implementation of corporate human rights accountability norms, and by proposing alternative conceptions of accountability.

The reconstruction of the creation of the UNGPs shows that it was the SRSG’s aim, after the failure of the UN Norms, to seek the broadest possible consensus among stakeholders, including business actors, while avoiding the imposition of new, direct, and legally binding obligations on corporations under international law (Hamm 2022; see also Buhmann 2013). The confined definition of corporate human rights accountability in the UNGPs as a non-binding responsibility to respect was thus the deliberate outcome of the “strategic social construction” (Finnemore and Sikkink 1998: 888) of a particular norm definition at the international level which (temporarily, at least) superseded other, potentially more restrictive, accountability models and instruments. The UNGPs’ definition of accountability as the responsibility to respect human rights requires business firms to “avoid infringing on the human rights of others” and to carry out “due diligence,” which means “to identify, prevent, mitigate and account for how they address their adverse human rights impacts.”^21^ Yet, this defensive conception of corporate human rights accountability “hardly exhausts” the potential of corporations “to contribute to a world more human rights-compliant” (Gregg 2021: 3), as the contributions in this special issue show both at a theoretical and empirical level. Pointing out the limits of the UNGPs’ do-no-harm approach, they propose more comprehensive and ambitious conceptions of human rights accountability which would encourage the transformation of the business models of conventional corporations and subject corporate sovereignty to external, democratic control (Gregg 2021). They explore different models of corporate human rights accountability and corresponding governance instruments to be implemented in a future binding treaty and develop an analytical framework for their critical assessment (Bernaz 2021).

### The Modes of Governance

Norm scholarship may also encourage us to investigate the *modes of governance* through which corporate human rights accountability norms are constructed, diffused, and transformed. This implies analyzing the networks of actors and institutions, as well as the interplay of the horizontal and vertical dimensions of governance processes. Due to the transnational character of economic activities and the involvement of public and private actors, the business and human rights field “re-draws the perimeter of the national human rights system” (Methven O’Brien and Ford 2019: 216), thereby challenging traditional state-centric paradigms of human rights scholarship. In fact, the UNGPs were deliberately designed so as to move beyond a narrow focus on state compliance and better align the three distinct governance systems shaping corporate conduct at the global level: the traditional system of national and international public law and governance; the system of civil governance involving a range of stakeholders and civil society actors; and the system of corporate governance which includes elements of the other two systems but consists primarily of the self-regulation of business (Ruggie 2020: 74). While the polycentric governance approach which underlies the UNGPs’ construction seeks to accommodate a variety of actors and institutions, as well as different forms of power in the business and human rights arena, scholars have pointed out that it leaves unanswered “questions of coherence, legitimacy and accountability” (Methven O’Brien and Ford 2019: 230).

The articles in this special issue address these gaps. They do so by highlighting the fundamental struggles in the business and human rights field over the power to define corporate human rights accountability and its adequate implementation, as well as over the legitimacy of the adequate mode of governance (Hamm 2022; Bernaz 2021). While the UNGPs’ proponents point to their broad diffusion and superior legitimacy derived from an inclusive multi-stakeholder consultation process, advocates of a binding international treaty call for legally enforceable standards and argue that legitimacy in international law cannot be generated through private governance mechanisms but can only be derived from negotiations between (democratically elected) governments (Hamm 2022). Moreover, the articles in this special issue reveal a structural misalignment in the governance architecture of the business and human rights regime with regard to the envisaged role of specific actors and their actual capabilities, as is the case with National Human Rights Institutions (Wolfsteller 2022). They point out significant gaps in the inclusiveness of participatory governance tools such as National Action Plans on business and human rights (Methven O’Brien et al. 2022). And they reveal the fortification of power and resource asymmetries between transnational corporations and victims of corporate human rights abuses, even through seemingly progressive legal instruments, such as the French Duty of Vigilance Law (Schilling-Vacaflor 2021).

### Effectiveness

Theories and concepts of norm scholarship can also help illuminate one of the largest and most persistent gaps in business and human rights research, which is the lack of knowledge about the *effectiveness of corporate human rights accountability norms and instruments*. We define effectiveness as comprising three dimensions: first, it refers to the acceptance and uptake of a norm by relevant actors in the field; second, it describes the extent to which a norm has triggered behavioral change; and, finally, it points to the intended as well as the unintended consequences of that behavioral change and regulatory interventions. As indicated above, norm scholarship’s critical awareness and useful conceptualization of the different outcomes of norm construction and diffusion processes may assist in decentering the legal perspective which still dominates business and human rights research.

The articles in this special issue seek to advance our understanding of the effectiveness of different regulatory instruments and key actors in the business and human rights regime by analyzing the factors that hinder or facilitate their capacity to bring about sustainable human rights change. They show that voluntary commitments by companies to respect human rights have only limited impact on their business practices because managers find it difficult to identify human rights indicators and to formulate a positive business case for human rights respect and promotion (Favotto and Kollman 2022). The effectiveness of legally binding home state regulations supposed to provide access to remedies for victims of corporate human rights abuses by subsidiary companies of TNCs suffers from the structural defect of placing the burden of proof on the side of the claimants, as the analysis of the French Duty of Vigilance Law demonstrates (Schilling-Vacaflor 2021). Similarly, the study of National Human Rights Institutions (NHRIs), which are envisaged by the UNGPs to fulfill the role of state-based, non-judicial grievance mechanisms in relation to corporate rights abuses, reveals that the majority of NHRIs has only limited powers and competences to provide access to remedies and pressurize corporate actors into human rights compliance (Wolfsteller 2022). While it is too early to assess the effectiveness of National Action Plans on business and human rights, the close examination of their construction processes shows a general lack of specific indicators and measurable targets, as well as a lack of monitoring capacities in many states which would allow for a meaningful evaluation and adjustment of NAPs in their reporting and revision cycles (Methven O’Brien et al. 2022).

## Outline of the Special Issue

This special issue begins with an article by Benjamin Gregg who addresses a fundamental problem of the business and human rights debate which is still both underexplored and undertheorized: the tension (or value conflict) between the logic of corporate profit-seeking in a globalized market economy and the normative aspiration of human rights as universalistic norms of moral justice and human dignity. In “Beyond due diligence: The Human Rights Corporation,” Gregg (2021)^22^ criticizes the UN Guiding Principles for their insufficient attempt to reconcile the self-seeking orientation of conventional corporations with the other-regarding logic of human rights. Proposing to move beyond the UNGPs’ defensive approach of corporate responsibility and human rights due diligence, Gregg develops a framework for transforming conventional corporations into “Human Rights Corporations.” A Human Rights Corporation would seek to fulfill the potential of business to contribute to human rights realization, not through mere avoidance of negative impact but by voluntarily and actively advancing and advocating human rights, with the encouragement of its shareholders and managers. In contrast to a conventional corporation, the corporate sovereignty of a Human Rights Corporation would be diminished to the extent necessary to prevent private property from harming the human rights of employees, stakeholders, and other persons affected by the company, according to Gregg’s conception. While the Human Rights Corporation would integrate, through voluntary embrace, human rights norms and values into its culture, strategy, and behavior, it would not completely abandon the aim of corporate profit-seeking but would pursue both aims as strategic priorities; otherwise, it would quickly cease to exist. To reinforce the transformation of conventional companies into Human Rights Corporations and to hold these to account, Gregg argues for the imposition of stronger frameworks for private sector regulation and accountability mechanisms subject to external, democratic procedures and control by the political community.

The importance of Gregg’s plea for pushing the boundaries of corporate engagement with human rights is underpinned by the analysis of corporate practice in the second contribution to this special issue. In their article “When rights enter the CSR field: British firms’ engagement with human rights and the UN Guiding Principles,” Alvise Favotto and Kelly Kollman (2022) investigate whether and how the corporate responsibility to respect human rights has been integrated into the business practices of TNCs and how it has affected their public commitments. Favotto and Kollman concentrate on TNCs headquartered in the UK which was the first country to adopt a National Action Plan on Business and Human Rights in 2013. Combining a longitudinal study of corporations’ CSR reports from 1995 to 2015 with original interview data, Favotto and Kollman find that firms have partially integrated human rights standards into their business practices and expanded the articulation of their responsibility for human rights following the adoption of the UNGPs in 2011 and the UK NAP in 2013. Yet, these changes remain largely limited to the defensive, do-no-harm approach proposed by the UNGPs and to selected procedural and managerial practices. There is no indication of a systematic assessment of the companies’ overall impact on human rights, no specific and comprehensive strategy for improving their human rights performance in the future, nor a transparent communication thereof to stakeholders. As business firms refrain, largely for strategic reasons, from showing their human rights impacts and future commitments, they “actively resist the scrutiny NGOs, international organizations and actors populating the CSR field demand,” as Favotto and Kollman (2022: 17) argue, which inhibits the UNGPs’ transformative potential. Only with respect to health and safety rights and diversity do companies take a more proactive role, both in terms of substantive behavioral changes and promotion. As the main reasons for the overall negligent corporate engagement with human rights, Favotto and Kollman identify the lack of insider champions, the absence of specific human rights indicators, and––above all––the perceived difficulty among CSR managers and investors to make a positive business case for human rights respect and promotion. The authors therefore argue for the establishment and diffusion of more rigorous reporting standards for companies’ human rights impacts and more stringent government regulation as introduced in other European countries.

In the following article “Putting the French Duty of Vigilance Law in context: Towards corporate accountability for human rights violations in the Global South?”, Almut Schilling-Vacaflor (2021)^23^ explores the potential and pitfalls of such stringent national regulations. She examines the applicability of the French Duty of Vigilance Law of 2017 to the activities of Total E&P, a subsidiary company of the French-based TNC Total, in indigenous territories of Bolivia’s Chaco region, in order to assess the extent to which legally binding home state regulations can break through the “separation principle” of dissociated accountability of parent and subsidiary companies for their human rights and environmental impacts. Schilling-Vacaflor draws on original material from a year of extensive fieldwork to show that the socio-environmental impact resulting from the subsidiary company’s exploration and gas production in Bolivia indeed negatively affects the rights of local indigenous communities. Through applying “divide and rule” tactics, Total E&P systematically divided these communities and undermined their right to free, prior, and informed consent (FPIC), and their right to fair compensation, water, and employment, thus bringing the company within the ambit of the French Duty of Vigilance Law. Yet, Schilling-Vacaflor’s analysis shows that the burden of proof on the side of the claimants to establish the facts of the rights abuse, and the structural divides in power and resources between transnationally operating business enterprises and indigenous communities in the Global South significantly diminish the ability of victims to take legal action in a foreign jurisdiction and seek redress under the Duty of Vigilance Law. Although the law formally established legal liability of TNCs for adverse human rights and environmental impacts of subsidiary companies operating abroad, Schilling-Vacaflor questions whether home state regulations modelled on the French Duty of Vigilance Law can indeed provide victims of corporate human rights abuse with effective access to remedy in practice.

In the fourth contribution, René Wolfsteller examines the role and effectiveness of another governance tool that the Guiding Principles and subsequent UN resolutions regard as a particularly promising instrument for the provision of access to remedy for victims of corporate human rights abuses: NHRIs. In “The unrealized potential of National Human Rights Institutions: Conditions for effective engagement and proposal for reform,” Wolfsteller (2022) identifies a broad legal mandate extending to the private sector and a combination of strong promotional and complaints handling powers as essential structural conditions for effective NHRI engagement with business-related human rights abuses. The fact that the Paris Principles as the most important international steering instrument for the design and functions of NHRIs do not prescribe these features contributed to a situation, according to Wolfsteller’s analysis, in which a significant number of institutions has no complaints handling powers in relation to business actors and only a small minority of NHRIs has been vested with powers and mandates covering both public and private actors and a broad range of rights norms. As a result, the majority of NHRIs is severely limited in their ability to improve human rights compliance in the private sector and, in particular, to fulfill the role of state-based, non-judicial grievance mechanisms in relation to corporate rights abuses as envisaged by the UNGPs. To resolve that mismatch, Wolfsteller proposes to adjust and apply the Paris Principles in a way which would increase the pressure on states to enhance the mandates and powers of their NHRIs with regard to the prevention and remedy of business-related human rights abuses. Revising the Paris Principles in this way, he argues, would not only contribute to the “hardening” of the UN Guiding Principles’ soft law provisions but would also strengthen NHRIs’ capability to function as national implementation mechanisms of a potential business and human rights treaty in the future.

In the following article “National Action Plans on Business and Human Rights: An experimentalist governance analysis,” Claire Methven O’Brien, John Ferguson and Marisa McVey (2022) analyze the development processes of National Action Plans on business and human rights (NAPs) in 25 states through the lens of experimentalist governance theory. Advanced in response to critics of the legitimacy and effectiveness of international human rights norms, experimentalist human rights governance takes into account and facilitates stakeholder participation and processes of collective learning and revision, which, according to the authors, renders it particularly suitable for application to business and human rights regulation. Indeed, Methven O’Brien, Ferguson, and McVey find that most NAP processes resemble key features of effective local implementation mechanisms hypothesized by experimentalist governance theory, such as stakeholder participation, agreement on a common problem, implementation, monitoring and reporting mechanisms, and procedures to evaluate the effectiveness of policy measures. However, in their study, the authors also identify weaknesses in states’ NAP development practice of the past and present. In most states, stakeholder consultations for NAP development did not sufficiently include vulnerable groups who are at heightened risk of being marginalized and discriminated against. Moreover, most NAPs are short of explicit indicators and measurable targets, with government administrations often lacking the institutional and informational apparatus to generate reliable data and monitor the implementation of policy measures. As a result, it is often unclear how processes of peer review-based evaluation and policy learning can be secured, and how NAPs can be meaningfully adjusted to increase their effectiveness. Nonetheless, the authors note that, despite significant differences in their structure and approach, NAPs have generated relatively sophisticated governance architectures. If future NAP development processes can remedy the weaknesses identified by Methven O’Brien, Ferguson, and McVey in their analysis, the authors are hopeful that NAPs can contribute to human rights change in the business sector.

The final two articles provide original analyses of corporate accountability, legitimacy, and governance aspects of a future binding treaty on business and human rights. In the contribution “Conceptualizing corporate accountability in international law: Models for a business and human rights treaty,” Nadia Bernaz (2021)^24^ seeks to bring conceptual clarity and normative ambition both to the treaty negotiations and to human rights scholarship about the substance of corporate human rights accountability. Bernaz conceptualizes corporate accountability in international law and develops an analytical framework which helps assess different conceptions in the debate. Based on seven indicators encompassing dimensions of actor type, answerability, and enforceability, Bernaz reconstructs four distinct models of corporate accountability from business and human rights scholarship. She identifies the UNGPs’ approach as arguably the weakest possible form, with corporate accountability defined as a social expectation of do-no-harm without binding obligations and sanctions. A second approach follows the Universal Declaration of Human Rights model of accountability and envisages business enterprises taking an active role in the protection and promotion of human rights yet without specific enforcement mechanisms. The third conception termed by Bernaz as the “progressive model” grounds corporate accountability in international law while relying on domestic enforcement mechanisms. The fourth approach––the “transformative model”––also derives its conception of corporate accountability from international law standards but advocates their enforcement by new international institutions and mechanisms, such as a tailor-made international criminal court. Against the backdrop of the relatively weak form of corporate accountability adopted in the current Draft Treaty, Bernaz makes a powerful case for adopting a more ambitious yet balanced approach in line with the “progressive model.” Corporate accountability so defined would reconcile human rights ideals with the practical demands of political realism, she argues, as it would impose direct human rights obligations on business firms but would leave their enforcement to the domestic level to secure the necessary state support.

Brigitte Hamm concludes this Special Issue with a comparative analysis of the development processes of the UN Guiding Principles and the Draft Treaty from October 2019 as negotiated by the OEIGWG. In “The struggle for legitimacy in business and human rights regulation: A consideration of the processes leading to the UN Guiding Principles and an international treaty,” Hamm (2022) argues that both processes epitomize fundamental struggles in the business and human rights field over the power to define corporate responsibility and its adequate implementation, and over the legitimacy of the adequate mode of governance. According to Hamm’s analysis of the debate, the authors and proponents of the UNGPs point to the Principles’ superior legitimacy derived from an inclusive consultation process with a broad range of stakeholders, while the advocates of a treaty argue that legitimacy in international law cannot be generated through private governance mechanisms but can only be derived from negotiations between (democratically elected) governments. Moreover, treaty advocates criticize the soft law approach of the UNGPs as insufficient and call for the imposition of legally binding and enforceable human rights obligations on corporations. Although Hamm shares the criticisms of the UNGPs––especially the underrepresentation of victims of corporate rights abuses in the consultations––she submits that the development process of the Draft Treaty suffered from serious limitations as well. In particular, Hamm calls for greater flexibility and reforms of the UN process so as to broaden participation by civil society actors and produce a treaty which fully accounts for the rights of victims. If the future treaty would establish legal liability of transnationally operating business enterprises, Hamm argues, the combination of soft and hard law instruments for the regulation of business and human rights may contribute to the creation of a level playing field in the global economy that may strengthen human rights in the long run.

## Conclusion and Avenues for Future Research

Taken together, the contributions to this special issue offer a rich multi-dimensional and transdisciplinary analysis of key elements of the business and human rights regime. On the one hand, they enhance our understanding of different conceptions of corporate human rights accountability, of the role and functions, the impact but also the limitations of important actors and governance instruments of that regime. On the other hand, the articles engage with these limitations in a critical yet constructive fashion, making proposals for resolving misalignments in the governance architecture and improving the effectiveness of specific instruments. In so doing, all contributions are driven by a normative commitment to strengthen corporate human rights accountability, help prevent business-related human rights abuses, and improve access to effective remedies for victims.

Building on the insights provided by the analyses in this special issue, as well as on the research agenda set out above, we identify three main tasks for future research in the business and human rights field. Firstly, scholarship needs to further investigate the scope and substance of changes in corporate human rights conduct in response to regulatory initiatives. This is not only to differentiate good corporate practice from rhetorical adaptation or strategic attempts to co-opt the human rights discourse but also to identify the underlying conditions for the effective socialization of business actors into norm acceptance and compliant behavior. Secondly, researchers need to keep pace with the evolution of, and changing trends in, the business and human rights regime. This implies the close monitoring and critical assessment of the construction, contestation, and transformation of human rights and accountability norms, as well as of the emergence and performance of actors and governance instruments, such as National Contact Points, National Action Plans, corporate reporting standards, and due diligence laws. Here, it is important to move beyond dominant perspectives on the Global North and extend the research focus to regulatory frameworks and corporate conduct at the starting point of transnational supply chains, especially in countries of the Global South. Finally, business and human rights scholars should continue to feed their academic expertise into the policy process and public debate at the local, national, and transnational levels. With their privileged access to the insights of multidisciplinary human rights scholarship, business and human rights scholars are in an ideal position to create awareness for the complexity of the issues at stake, help building bridges between opposing actors, and contribute to the enhancement of corporate human rights accountability.

## Footnotes

1 United Nations Human Rights Council (UNHRC), A/HRC/RES/17/4, 6 July 2011, para. 1.

2 For a recent case analysis of the garment industry in the context of the COVID-19 pandemic, see Li et al. 2021.

3 It is highly controversial among legal scholars whether private business enterprises are subjects of public international law and, in particular, if they have direct obligations under international human rights law. For a summary of the debate and a progressive position, see Bernaz 2017: 86–101.

4 In fact, many western liberal democracies conceptualize human rights as a product that is primarily “for export” to other countries and tend to contest their stronger domestic institutionalization. For the detailed analysis of an exemplary case of that western hubris, see Wolfsteller 2020 on the failed translation of international human rights norms in the UK.

5 E/CN.4/Sub.2/2003/12/Rev.2, 26 August 2003.

6 Ruggie 2013: xxx-xxxii. Relevant regulatory instruments include, for instance, the ILO Declaration on Fundamental Principles and Rights at Work 1998, the EU Timber Regulation 2010, the EU Conflict Minerals Regulation 2017; the U.S. Dodd-Frank Wall Street Reform and Consumer Protection Act of 2010 which, in section 1502, regulates companies’ disclosure of the use of conflict minerals; the UK Modern Slavery Act 2015; and the Australia Modern Slavery Act 2018. With respect to the USA, the Alien Tort Statute provided a useful tool for civil litigation against public and private actors in human rights cases for many years, although its scope and applicability were significantly narrowed by decisions of the U.S. Supreme Court in 2013 and 2018, effectively barring claims against foreign corporations. For a detailed discussion of “the rise and fall” of the ATS, see Stephens 2020.

7 The PRR framework is set out in Ruggie’s 2008 report to the UN: UNHRC A/HRC/8/5, 7 April 2008.

8 See the Guiding Principles in the Annex to Ruggie’s final report to the UN Human Rights Council in: UNHRC A/HRC/17/31, 21 March 2011.

9 UNHRC A/HRC/RES/17/4, 6 July 2011, para. 1.

10 E.g., the EU’s Non-Financial Reporting Directive, Directive 2014/95/EU, with corresponding guidelines, available at: https://ec.europa.eu/info/publications/non-financial-reporting-guidelines_en; and the EU’s CSR strategy, see Communication from the Commission to the European Parliament, the Council, the European Economic and Social Committee and the Committee of the Regions (2011), A Renewed EU Strategy 2011–14 for Corporate Social Responsibility, available at: http://eur-lex.europa.eu/legal-content/EN/TXT/?uri=celex%3A52011DC0681 (all accessed 30 September 2021).

11 OECD Guidelines for Multinational Enterprises 2011, available at: https://www.oecd.org/daf/inv/mne/48004323.pdf (accessed 30 September 2021).

12 See, e.g., International Organisation of Employers (IOE), International Chamber of Commerce (ICC) and the Business and Industry Advisory Committee (BIAC) to the OECD, Joint statement on business and human rights to the United Nations Human Rights Council Geneva, 30 May 2011; China Chamber of Commerce of Metals, Minerals and Chemicals Importers and Exporters, Guidelines for social responsibility in outbound mining investments, 2014; see also the human rights policies by TNCs such as the Volkswagen AG, available at https://www.volkswagenag.com/en/sustainability/business-and-human-rights.html#; by Royal Dutch Shell, available at: https://www.shell.com/sustainability/communities/human-rights/_jcr_content/par/textimage_432255052_.stream/1620319789073/97788a0ea7c09cc6c63dece84a6d508947030e68/shell-pp-human-rights-may.pdf; and by Nestlé, available at: https://www.nestle.com/csv/what-is-csv/ethical-business/human-rights (all accessed 30 September 2021).

13 E.g., ISO26000, a guidance document on social responsibility by the International Organization for Standardization; the International Finance Corporation’s Performance Standards on Environmental and Social Sustainability, 1 January 2021.

14 By the end of 2020, 25 states had published National Action Plans on business and human rights. Between 2011 and the end of 2020, 42 NAPs on business and human rights were adopted or have been in development (Methven O’Brien et al. 2022, this issue).

15 See the OECD Guide for National Contact Points on Structures and Activities, 2019, available at: https://mneguidelines.oecd.org/Guide-for-National-Contact-Points-on-Structures-and-Activities.pdf (accessed 30 September 2021).

16 Some of the most recent human rights due diligence/supply chain regulations include: UK Modern Slavery Act 2015, EU Conflict Minerals Regulation 2017, French Duty of Vigilance Law 2017, Dutch Child Labor Due Diligence Law 2019, and German Act on Corporate Due Diligence in Supply Chains 2021. For a detailed analysis of due diligence laws and policies in Europe, see Schilling-Vacaflor and Lenschow (2021).

17 The open-ended intergovernmental working group on transnational corporations and other business enterprises with respect to human rights (OEIGWG) was established by the UNHRC with the mandate “to elaborate an international legally binding instrument to regulate, in international human rights law, the activities of transnational corporations and other business enterprises,” A/HRC/RES/26/9, 14 July 2014, para. 1.

18 The third revised draft of a treaty on business and human rights, 17 August 2021, is available at: https://www.ohchr.org/Documents/HRBodies/HRCouncil/WGTransCorp/Session6/LBI3rdDRAFT.pdf (accessed 30 August 2021).

19 These questions are raised and discussed with a special focus on the business case for human rights by, e.g., Li and McKernan (2017: 265); as well as Baumann-Pauly and Posner (2016). In light of historical examples of the involvement of corporations in gross human rights violations, genocide, and crimes against humanity, such as the Atlantic slave trade and the Holocaust, Bernaz (2017: 8) convincingly argues that “the search for a business case to respect human rights is inappropriate.”.

20 The analysis of corporate human rights due diligence practices by McCorquodale et al. (2017) provides an important starting point but only covers the perspective of business actors, not of the rights-holders affected.

21 UNHRC A/HRC/17/31, 21 March 2011, Annex, Guiding Principle 11 at p. 13, and Guiding Principle 17 at p. 16.

22 This article was intended for inclusion in this special issue. It was, however, first published in a separate issue, Vol. 22, Issue 1, and is available at: https://doi.org/10.1007/s12142-020-00605-x.

23 This article was intended for inclusion in this special issue. It was, however, first published in a separate issue, Vol. 22, Issue 1, and is available at: https://doi.org/10.1007/s12142-020-00607-9.

24 This article was intended for inclusion in this special issue. It was, however, first published in a separate issue, Vol. 22, Issue 1, and is available at: https://doi.org/10.1007/s12142-020-00606-w.
